# Step-up fecal microbiota transplantation strategy: a pilot study for steroid-dependent ulcerative colitis

**DOI:** 10.1186/s12967-015-0646-2

**Published:** 2015-09-12

**Authors:** Bota Cui, Pan Li, Lijuan Xu, Youquan Zhao, Huiquan Wang, Zhaoyuan Peng, Hai’e Xu, Jie Xiang, Zhi He, Ting Zhang, Yongzhan Nie, Kaichun Wu, Daiming Fan, Guozhong Ji, Faming Zhang

**Affiliations:** Medical Center for Digestive Diseases, The Second Affiliated Hospital of Nanjing Medical University, 121 Jiangjiayuan, 210011 Nanjing, China; Collega of Precision Instrument and Opto-electronics Engineering, Tianjin University, 92 Weijin Road, 300072 Tianjin, China; Biomedical Engineering Department, School of Electronics and Information Engineering, Tianjin Polytechnic University, 399 Binshui West Street, 300378 Tianjin, China; Clinical Nutrition, The Second Affiliated Hospital of Nanjing Medical University, 121 Jiangjiayuan, 210011 Nanjing, China; State Key Laboratory of Cancer Biology and Xijing Hospital of Digestive Diseases, The Fourth Military Medical University, 710032 Xi’an, China

**Keywords:** Steroid-dependent ulcerative colitis, Step-up fecal microbiota transplantation strategy, Microbiota

## Abstract

**Background:**

The strategy of using fecal microbiota transplantation (FMT) for refractory ulcerative colitis (UC) remains unclear if single FMT failed to induce remission. This study aimed to evaluate the efficacy and safety of a designed step-up FMT strategy for the steroid-dependent UC.

**Methods:**

Fifteen patients with steroid-dependent UC were enrolled, and treated with step-up FMT strategy. Follow-up clinical data was collected for a minimum of 3 months. Fecal microbiota composition before and post FMT of patients and related donors were analyzed by 16S rRNA sequencing.

**Results:**

Eight of fourteen (57.1 %) patients achieved clinical improvement and were able to discontinue steroids following step-up FMT. One patient was lost to follow-up. Among the 8 patients who responded, five (35.7 %) received one FMT therapy, one (7.1 %) received two FMTs, and two (14.2 %) received two FMTs plus a scheduled course of steroids. Four (28.6 %) of the 8 patients who responded maintained long-term remission during follow-up (3–18 months). Six patients (42.9 %) failed to meet the criteria of clinical improvement and maintained steroid dependence, though three experienced transient or partial improvement. Microbiota analysis showed that FMT altered the composition greatly, and a microbiota composition highly similar to that of the donor emerged in the patients with successful treatment. No severe adverse events occurred during treatment and follow-up.

**Conclusions:**

Step-up FMT strategy shows promise as a therapeutic strategy for patients with steroid-dependent UC, likely due to the successful restructuring of gut microbial composition.

Trial registration: ClinicalTrials.gov, Number NCT01790061

**Electronic supplementary material:**

The online version of this article (doi:10.1186/s12967-015-0646-2) contains supplementary material, which is available to authorized users.

## Background

Ulcerative colitis (UC) is a chronic idiopathic inflammatory bowel disease characterized by continuous mucosal inflammation with a relapsing and remitting course [1, 2]. Inducing and maintaining remission on a long-term basis are the primary goals of treatment. Corticosteroids are among the most effective therapies for patients with moderate to severe UC [1, 3]. In the Olmstead County cohort of IBD patients, up to forty percent of these patients require steroids to control symptoms, and at 1 year follow-up approximately twenty percent of patients remained steroid dependent [4]. Unfortunately, there are many undesirable side effects of steroids, such as infections, hyperglycemia, and bone loss, just to name a few. Thus, corticosteroid dependence in patients with UC is an important clinical problem and maintenance of steroid-free remission is a key treatment goal. Consensus guidelines recommend thiopurines as first line steroid-sparing therapy [5, 6]. Biologics therapies, such as anti-TNF medications [7, 8], are also effective, though the high costs of such therapies can be prohibitive. Fecal microbiota transplantation (FMT), a concept which originated in China a millennia ago [9], involves infusing healthy donor microbiota into the intestines of a patient to restore the intestinal microbiome. Early studies have suggested some therapeutic effect (of a single FMT) in the treatment of inflammatory bowel disease (IBD) including UC and Crohn’s disease (CD) [10–17]. However, response rates have not been as impressive as FMT for the indication of recurrent *Clostridium difficile* colitis where cure rates approach 90 % [18]. We therefore hypothesize that repeat FMT, in conjunction with a short course of corticosteroids may lead to better efficacy than a single FMT or corticosteroids for treatment of UC.

In 2012, we established a standardized FMT protocol and clinical work flow to overcome challenges in the purification of fecal microbiota from feces and to optimize clinical decisions on when or how to use FMT to treat IBD [13, 15]. Our previous study showed that the rate of clinical improvement and remission in CD patients (based on Harvey Bradshaw Index score) following a single FMT at the first month was 86.7 and 76.7 % respectively [13].Though there was partial response in patients with CD, a single FMT seemed to have limited efficacy for steroid dependent patients. Therefore, based on our results of single FMT for selected refractory CD [13] and our initial experience using FMT to treat UC (clinical trial NCT01790061), we designed a protocol of step-up FMT strategy for the treatment of steroid-dependent UC. This study aimed to evaluate the efficacy and the safety of step-up FMT for steroid-dependent UC and to identify the composition of fecal microbiota related to sustained clinical response.

## Methods

### Recruitment of patients and donors

A prospective observational study as a part of clinical trial (NCT01790061) was carried out by the Medical Center for Digestive Diseases at the Second Affiliated Hospital of Nanjing Medical University, Nanjing, China, from November 2012 to August 2014. Informed consent was obtained from all participants. The diagnosis of UC was established by a combination of typical clinical, endoscopic, and histological criteria. All included patients, had moderate to severe (S2 and S3) UC based on the Montreal classification [19]. Steroid dependence was defined as either the inability to reduce steroid dose below the equivalent of prednisone 10 mg/day within 3 months of starting steroids without recurrent active disease, or relapsing within 3 months of stopping steroids in accordance with ECCO guidelines [6]. Exclusion criteria were: severity of Montreal classification below S2; accompanied with other severe disease, including other intestinal diseases e.g. *C. difficile* infection, diabetes, cancers, and follow-up of less than 3 months.

Stool donors were selected according to the selection criteria described in our previous report [13]. Briefly, healthy children, age 10–17 years, were selected from patients’ relatives or friends, and were carefully screened by the following exclusion criteria: history of drug use, history of disease (e.g. antibiotic, laxative or diet pill use within the past 3 months; prior immunomodulator or chemotherapy use; history of all known infectious diseases, morbid obesity, diabetes, IBD, irritable bowel syndrome, chronic diarrhea, constipation, colorectal polyps or cancer, immunocompromised states, metabolic syndrome, allergy, chronic fatigue syndrome, history of major gastrointestinal surgery or systemic autoimmunity, as well as any other diseases or conditions potentially associated with changes in intestinal microbiota). In addition, all patients underwent laboratory evaluation including blood test (complete blood count), C-reactive protein, erythrocyte sedimentation rate, biochemical tests, Hepatitis A IgM, Hepatitis B surface antigen, Hepatitis B core IgG and IgM, antibodies, Hepatitis C antibody, human immunodeficiency virus types 1 and 2 antibody, Syphilis), and stool testing (i.e. stool culture, stool ova and parasites). Patients with any laboratory abnormalities were excluded.

### Purification of fecal microbiota and FMT

**Fig. 1:**
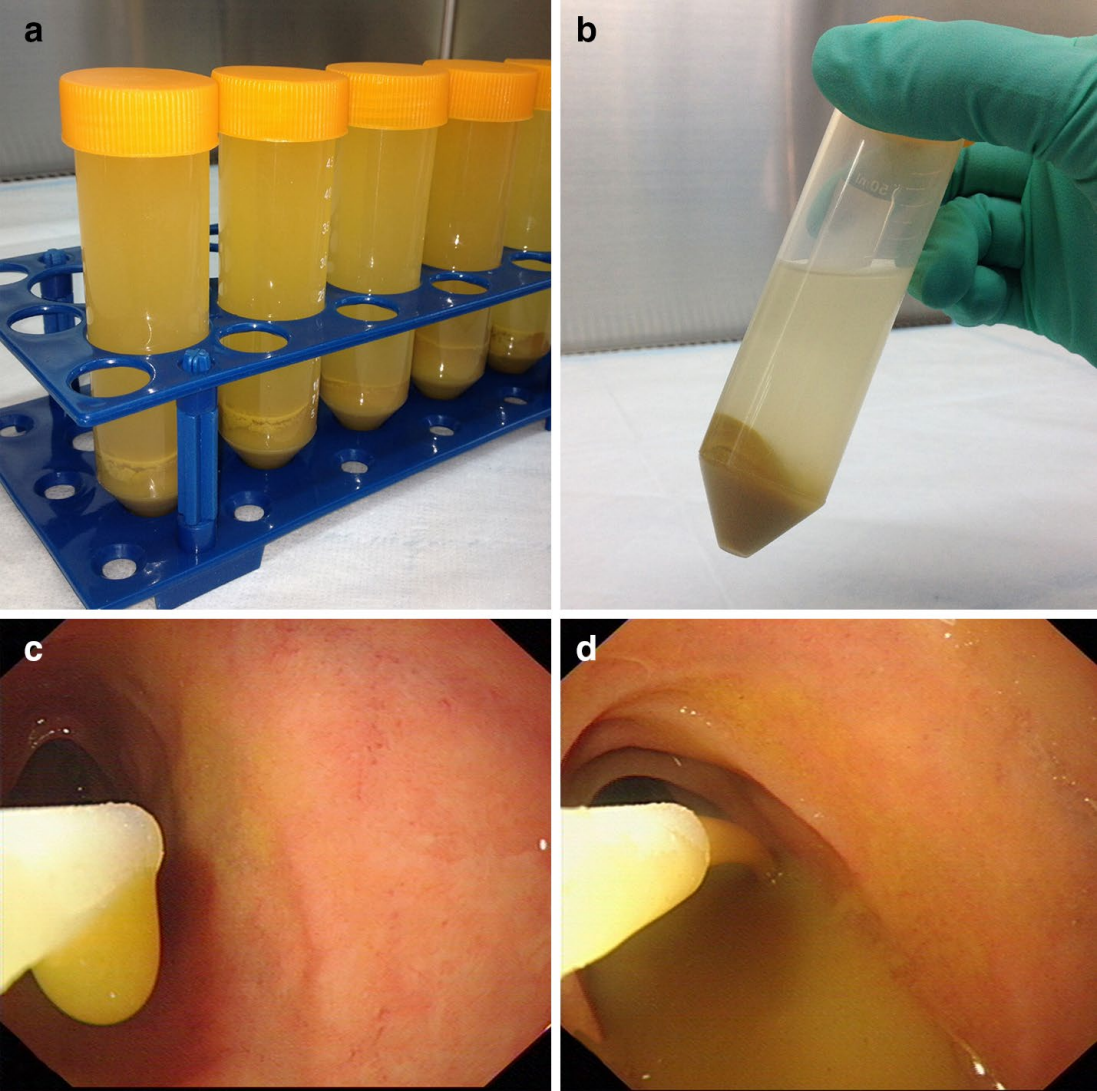
Laboratory enriched fecal microbiota and infusion of fecal microbiota during endoscopy. **a** The centrifuged microbiota in lab after microfiltration. **b** The final product for infusion. **c**, **d** The endoscopic image during infusion showing no observable particles in the suspension fluid under magnified endoscopic view, indicating the effect of purification for fecal microbiota

The fecal microbiota from donors was purified in our laboratory according to our previously published method of Filtration plus Centrifugation (FPC) [13]. Four of 14 patients underwent FMT based on a new developed automatic purification system (GenFMTer, FMT Medical, Nanjing, China). We named this new method for enriching microbiota based on an automatic purification system microfiltration plus centrifugation (MPC). Prepared microbiota was injected into the distal duodenum of recipients through an endoscopic infusion tube (FMT Medical, Nanjing, China) inserted into the gastroscope channel. The final enriched microbiota in lab and the endoscopic image during infusion were shown in Fig. 1. One hour prior to FMT, patients were given metoclopramide 10 mg by intramuscular injection and esomeprazole magnesium 40 mg intravenously to promote motility of the transplanted microbiota into the colon and to inhibit the secretion of gastric acid [13].

### Step-up FMT strategy

**Fig. 2:**
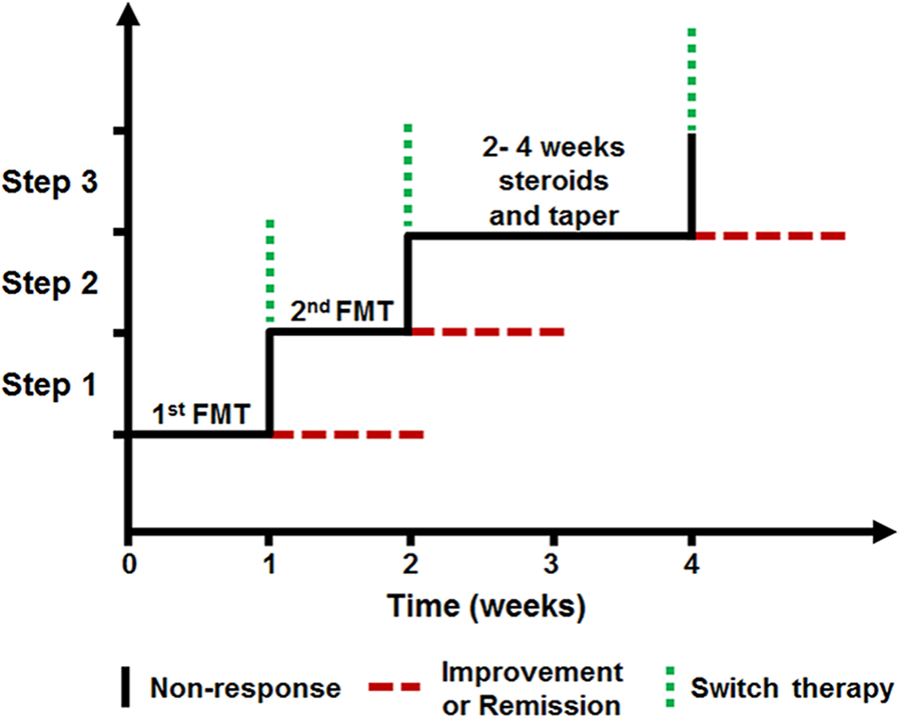
Flow chart of step-up FMT for steroid-dependent UC. The strategy includes three steps: *1* the initial FMT; *2* a second FMT after 1 week; *3* one short course of steroid therapy. Patients who fail to benefit from step 1 are advised to receive the second FMT. Patients who still had no response were switched to one-phase therapy using steroids. Withdrawal of corticosteroid started after 2–4 weeks of full dose of steroids. The patients who failed to benefit from each step could also choose biologic therapy or resumption of steroid therapy

The step-up FMT strategy was shown in Fig. 2. Before FMT, patient characteristics and baseline condition were assessed thoroughly. Steroid medications were tapered off at least 1 week prior to the first FMT, and other medication was also stopped. Patients were encouraged to eat a diet which limited animal protein, especially red meats, spicy foods, and high fat food. A low residue diet was encouraged. Mesalamine 3.0 g was given daily by oral as a sustained treatment before and after FMT [13]. Blood and stool samples were collected before and after FMT. All patients were followed for more than 3 months after the final scheduled FMT.

#### Efficacy and safety assessment

One week after FMT patients were assessed by symptoms such as abdominal pain, stool frequency, bloody purulent stool, as well as laboratory tests including erythrocyte sedimentation rate (ESR), C-reactive protein (CRP), basic chemistries, lymphocytes subset analysis, IgG, IgA and IgM. Clinical remission was defined as the absence of diarrhea and blood (Montreal classification S0); clinical improvement was defined as improvement in the severity of Montreal classification by more than one grade, e.g. from S3 to S2 or S1 [7]. Sustained clinical response was defined as a persistent steroid-free clinical improvement during follow-up, which was evaluated only in patients who achieved short-term clinical benefit. Steroid free was defined as absence of symptoms of active disease (no abdominal pain, no diarrhea, and no bloody purulent stool) without steroids within the next 3 months after step-up FMT. Safety was evaluated in all patients by recording adverse events during FMT and throughout long-term follow-up.

#### Stool sample collection and bacterial community analysis

Fecal samples from patients and donors were collected and stored at −80 °C for microbiota analysis by 16 s rRNA sequencing (Illumina, San Diego, CA, USA). Composition of fecal bacteria was analyzed at the phylum and genus level. Shannon’s diversity index was used to indicate the diversity of microbiota. Pearson correlation coefficient and principal coordinate analysis (PCoA) were used to indicate the similarity of microbiota composition among samples.

#### Statistical analysis

Data were analyzed by using SPSS (Chicago, IL, USA) or GraphPad (La Jolla, CA, USA). Analyses included paired student’s t test for paired data. Two tailed P value was calculated with each test. P values <0.05 were considered significant.

## Results

### Patient characteristics

A total of 15 patients (Table 1) with steroid-dependent active UC (mean 31.7 year-old, range 11–48 years, male/female 11/4) were enrolled. Mean disease duration was 4.3 years (range 1–9 years), 13 (86.7 %) of the patients had severe (S3) disease and 2 (13.3 %) had moderate (S2) UC severity. Eleven (73.3 %) patients had extensive lesions. Twelve (80.0 %) patients were being treated with other IBD medications besides steroids, such as 5-ASA and thiopurines.

**Table 1:** Patients characteristics, efficacy of step-up FMT strategy and medication before and after step-up FMT

Pt	Age (year)	Sex	Duration (year)	Location/severity	Medication before step-up FMT		FMT strategy	Clinical response to FMT	Medication after step-up FMT	Free of steroid
					Status of steroids usage (equivalent to prednisone, mg/day)	Combination therapy				
1	11	M	1	Left sided/S3	Maintenance dose 10 mg/day	Mesalamine 3.0 g/day	One FMT	No response	Prednisone 10 mg/day as maintenance and then switch to anti-TNF therapy for half a year	No
2	48	F	1	Extensive/S3	Relapse after withdrawal of prednisone to 10 mg/day	AZA 50 mg/day	One FMT	Short-term improvement and flared within 1 month	Prednisone 15 mg/day as maintenance	No
3	47	M	9	Extensive/S3	Relapse after withdrawal of prednisone	AZA 50 mg/day	One FMT	Short-term improvement and flared within 1 month	Prednisone 10 mg/day as maintenance	No
4	19	F	4	Extensive/S3	Maintenance dose 10 mg/day	Mesalamine 3.0 g/day	One FMT	No response	Switch to anti-TNF at the first week	No
5	20	F	6	Extensive/S3	Maintenance dose 20 mg/day for 3 years before FMT	Mesalamine 3.0 g/day	One FMT	Short-term improvement and flared within 1 month	Prednisone 20 mg/day as maintenance	No
6	32	M	1	Extensive/S3	Maintenance dose 10 mg/day	Mesalamine 4.0 g/day	One FMT	S3 to S1	Mesalamine 3.0 g/day as for 4 months	Yes
7	32	M	8	Extensive/S3	Serious flare with shock and diarrhea after withdrawal of prednisone to 20 mg/day	Mesalamine 4.0 g/day	Two FMTs	S3 to S2	Scheduled prednisone and then switch to Mesalamine 3.0 g/day for 4 months	Yes
8	34	M	4	Extensive/S3	Relapse immediately after withdrawal of prednisone	TCM	One FMT	S3 to S2	Mesalamine 3.0 g/day for 5 months	Yes
9	23	M	2	Extensive/S3	Maintenance dose 10 mg/day for 7 months	Mesalamine 4.0 g/day	Two FMTs	S3 to S2	Mesalamine 3.0 g/day for 6 months	Yes
10	38	M	8	Extensive/S3	Maintenance dose 10 mg/day for more than 2 years	AZA 50 mg/day	One FMT	S3 to S0	Mesalamine 3.0 g/day for 9 months	Yes
11	39	M	7	Left sided/S2	Relapse immediately after withdraw of prednisone to 15 mg/day	–	One FMT	S2 to S0	Mesalamine 3.0 g/day for 18 months	Yes
12	37	F	3	Proctitis/S2	Maintenance dose 35 mg/day for 31 months	Mesalamine 4.0 g/day	One FMT	S2 to S0	Mesalamine 3.0 g/day for 11 months	Yes
13	35	M	1	Extensive/S3	Maintenance dose 20 mg/day for 3 years and 2 months	Mesalamine 4.0 g/day	Two FMTs	S3 to S0	Follow by scheduled prednisones and then switch to Mesalamine 3.0 g/day as maintenance without relapse for 12 months	Yes
14	27	M	2	Extensive/S3	Relapse after withdrawal of prednisone to 15 mg/day	–	Two FMTs	Short-term improvement but flared soon afterward. Long-term remission of multiple skin infections	Prednisone 15 mg/day as maintenance	No
15	33	M	6	Left sided/S3	Maintenance dose 10 mg/day for 2 years	–	One FMT	S3 to S2, but lost to follow-up	–	–

*Pt* patient, *M* male, *F* female, *AZA* azathioprine, *TCM* traditional Chinese medicine

### Cases serials of FMT

Clinical efficacy of step-up FMT is shown in Table 1. One patient (patient 15) was lost for follow-up. Of the remaining 14 patients, 8 (57.1 %) achieved clinical improvement and could discontinue steroids following step-up FMT. Among these 8 patients, five (35.7 %) received one FMT therapy, one (7.1 %) received two FMTs, and two (14.2 %) received two FMTs plus one scheduled therapy with steroids. Four (28.6 %) of these 8 patients maintained long-term remission during follow-up (3–18 months). 42.9 % (6/14) patients failed to meet criteria for clinical improvement or clinical remission and continued to be steroid dependent, though some experienced a transient improvement or partial decrease in symptom severity.

Two patients (patient 1 and 4) chose to switch therapy for being nonresponsive to the first FMT. Three (patient 2, 3, and 5) had an immediate response to the initial FMT but flared within 1 month and then chose to be treated with steroids afterwards.

Four patients (patient 7, 9, 13 and 14) received the second FMT due to limited efficacy of the first FMT. Patient 7 had limited response after two FMTs; however he benefited from the steroid treatment after the second FMT and was able to withdraw from the steroids successfully. His disease flared after being maintained for 4 months’ improvement (S3 to S2); however the severity of relapse was lower than before FMT. Patient 9 achieved clinical improvement (S3 to S2) 7 days after the second FMT, which was maintained for 6 months. He then changed to TCM (Traditional Chinese medicine) therapy. Patient 13 had no response to the first FMT, and his second FMT was postponed by 3 weeks to allow for drainage of a worsening perianal abscess (not related to FMT). After the second FMT, he benefited from the scheduled steroid therapy and maintained S1 or S0 disease severity for more than 12 months. Patient 14 had clinical improvement (S3 to S1) after two FMTs and achieved the remission of multiple skin lesions which was related to impaired immunity by long-term steroid use. He was given a scheduled course of steroid therapy not because of his intestinal symptoms, but for fatigue.

### Response to FMT

**Fig. 3:**
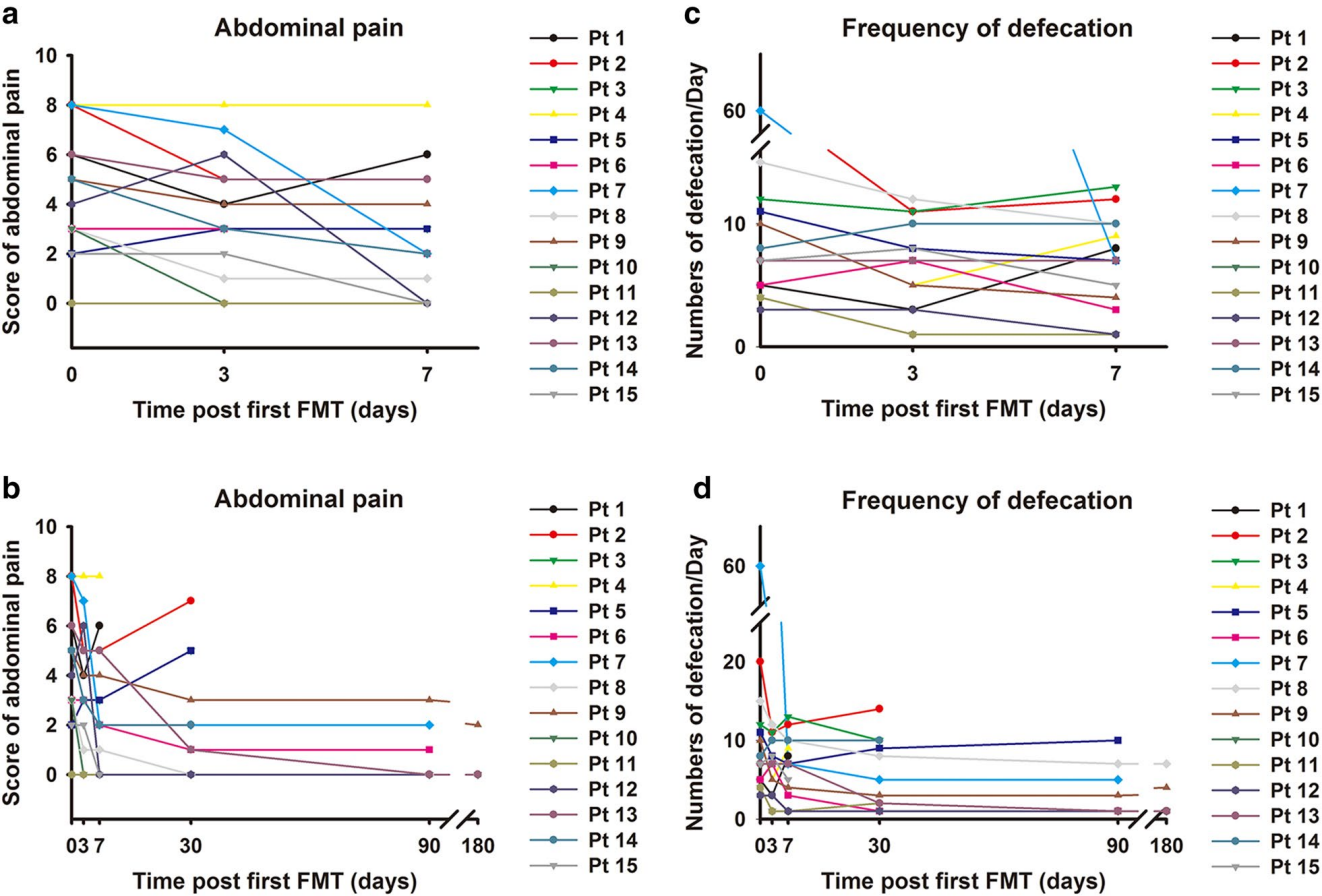
Clinical response to step-up FMT. **a** Abdominal pain scores of patients with steroid-dependent UC at baseline and the first week after initial FMT (n = 15). 10 patients showed significant improvement of abdominal pain after FMT. **b** Abdominal pain score at baseline and throughout follow-up after step-up FMT (n = 15); **c** frequency of patients’ defecation before and at 1 week after initial FMT (n = 15), 12 patients showed improvement in stool frequency; **d** change in defecation frequency at baseline and throughout follow-up after step-up FMT (n = 15)

As shown in Fig. 3, the overall score for abdominal pain and stool frequency decreased after FMT. Patients who experienced disease flare within 1 month of FMT had CRP, ESR, white blood cells (WBC) and lymphocyte subgroup populations that did not change following FMT (Additional file 1: Table S1). In contrast, patients who achieved clinical improvement and clinical remission had significantly lower ESR than those without response to FMT (*t test*, *P* < *0.05*). CRP also showed a similar trend, but no statistical difference was observed (Additional file 1: Table S1).We recommended follow-up endoscopy at our center at 3 or 6 months after step-up FMT. However, two cases refused due to symptoms and six due to the long distance to our hospital from their home. Thus only five cases underwent colonoscopy at 3 months after step-up FMT. In all five cases, the severity of colitis was markedly improved after FMT, but the improvement was not reflected by Mayo score. Therefore, based on the preliminary FMT experience and ethic consideration, we did not suggest each patient to accept colonoscopy 3 months after FMT.

### Fecal microbiota analysis

The changes of intestinal microbiota were analyzed in fecal samples from four patients (patient 6, 9, 10, and 13) and their related donors (patient 6 and 10 shared the same donor).These patients achieved clinical improvement and were steroid-free after therapy. The other patients came from all over the entire nation of China, and it was therefore inconvenient for them to re-visit, hence fecal samples post-FMT could not be obtained in our lab.

#### The alteration of microbiota in steroid-dependent UC patients

**Fig. 4:**
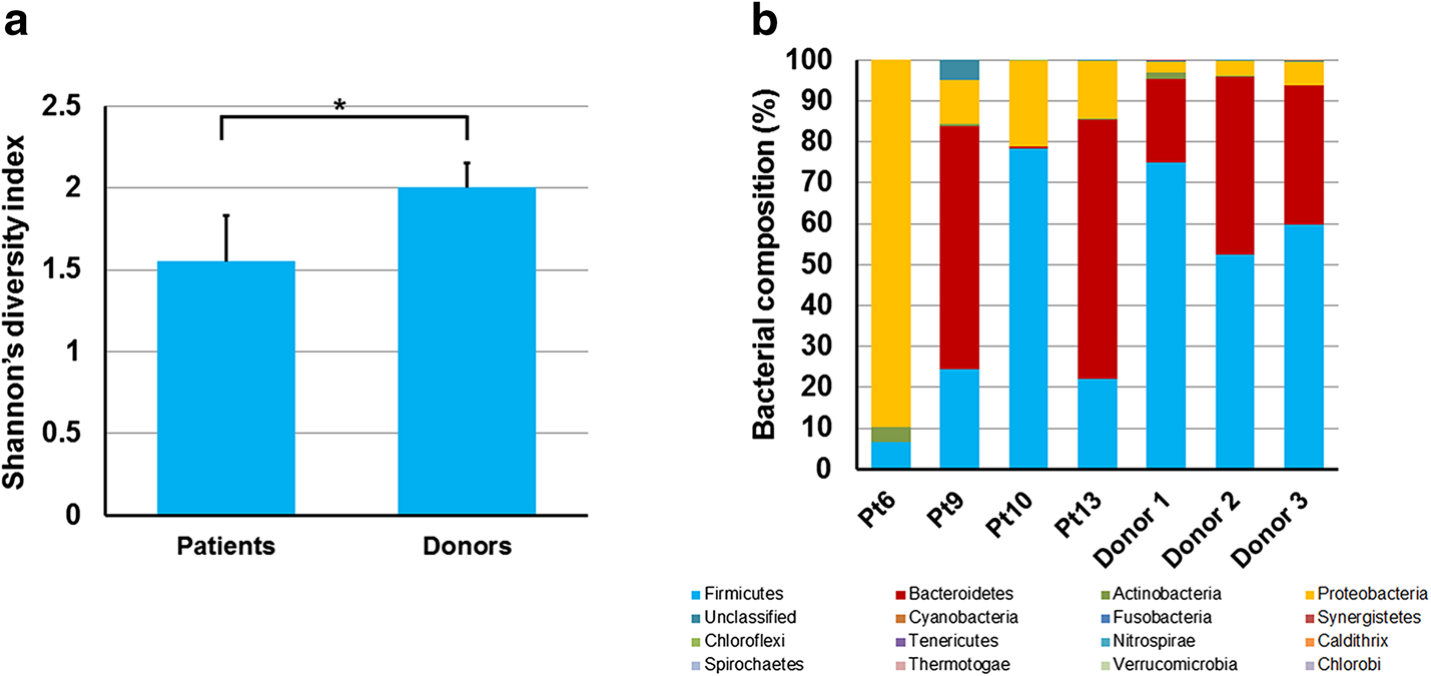
Analysis of fecal microbiota of patients with steroid-dependent UC. **a** The diversity of fecal microbiota (Shannon’s diversity index) showed significant decrease in steroid-dependent UC compared with healthy donors (*P < 0.05); **b** fecal microbiota composition at the phylum level in patients with steroid-dependent UC and in healthy donors

As shown in Fig. 4a, prior to FMT, the diversity of fecal microbiota of patients (n = 4) was decreased compared to donors (n = 3) (P = 0.004). Figure 4b demonstrates the composition of fecal microbiota based at the phylum level. The major bacteria of the donors were classified as *Firmicutes* (62.3 ± 11.5 %) and *Bacteroidetes* (32.7 ± 11.6 %), which comprised 95 ± 1.1 % of the total fecal microbiota. The characteristics of bacterial composition in UC patients showed an imbalanced ratio of *Firmicutes* and *Bacteroidetes* and an increase of *Proteobacteria*.

#### Change of intestinal microbiota after FMT

**Fig. 5:**
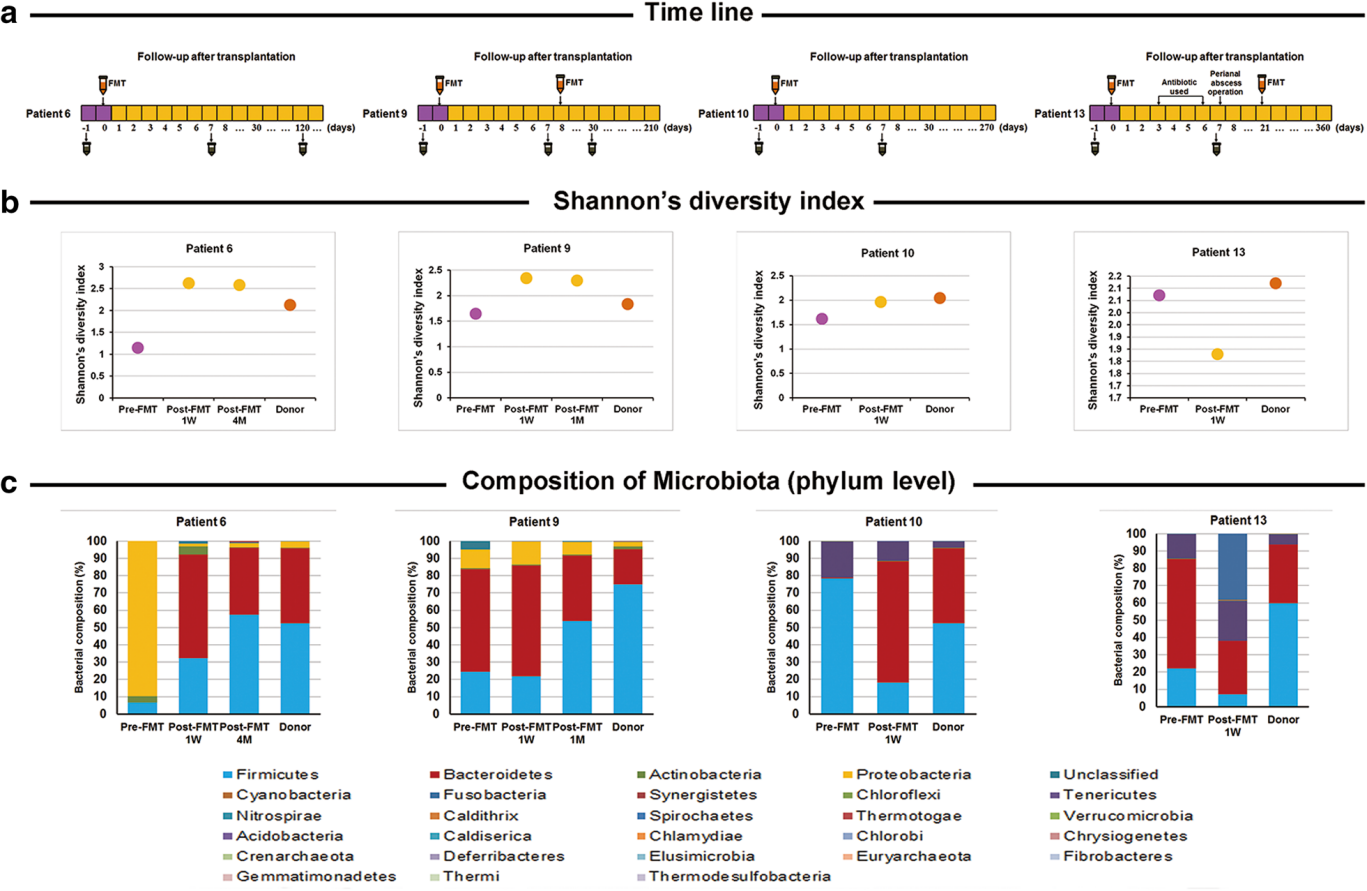
Change of fecal microbiota composition after FMT at a phylum level. **a** The schedule of fecal sample collection; **b** change in Shannon’ diversity index before and after FMT; **c** analysis of fecal microbiota composition at the phylum level before and after FMT

Figure 5a indicated the time-point of stool sample collected, and Fig. 5b showed the change of the bacterial diversity after FMT. The bacterial diversity after FMT increased in patient 6, 9 and 10, and was reduced in patient 13, likely due to antibiotics used before the second FMT.

**Fig. 6:**
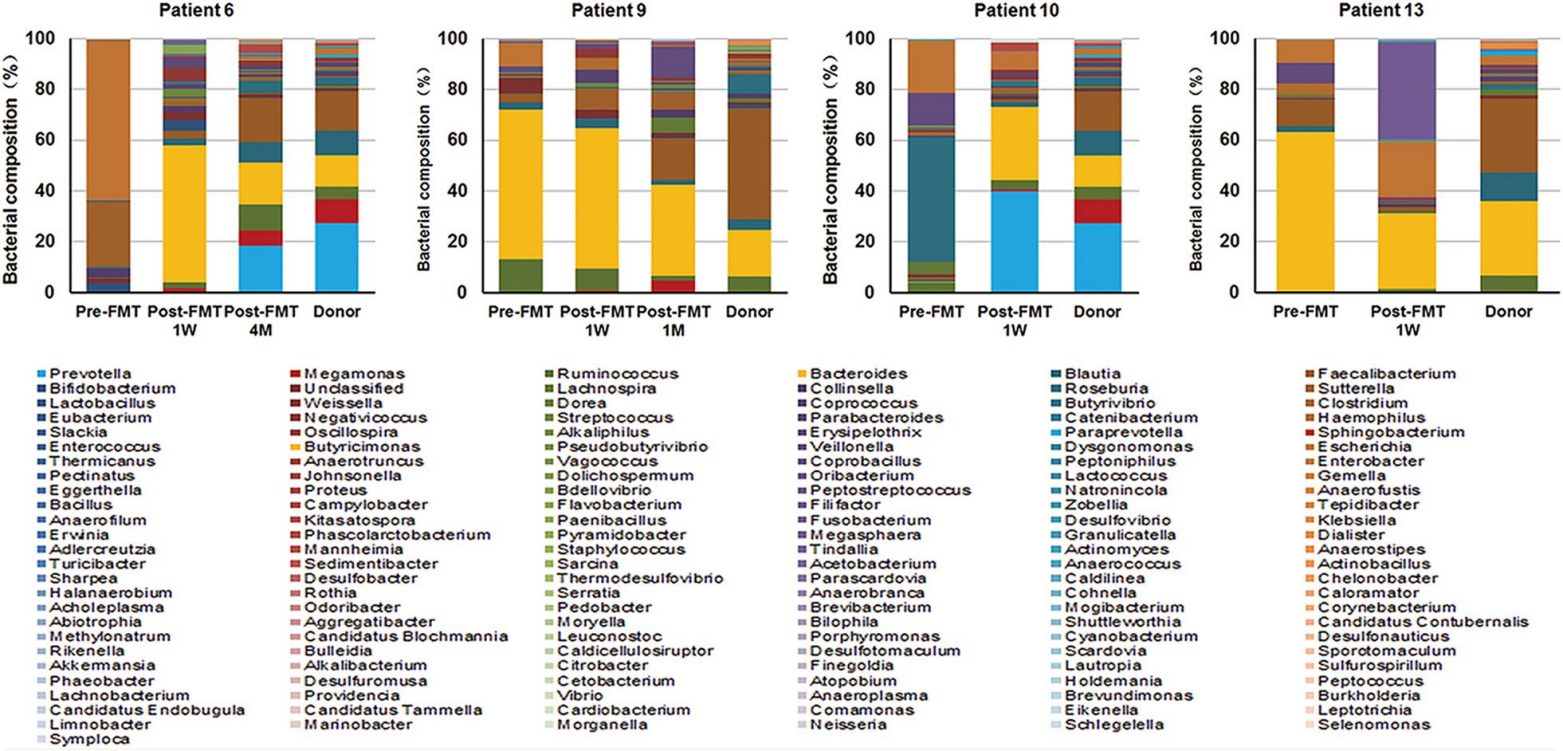
Genus level change in fecal microbiota composition following (two) FMTs in ulcerative colitis patients who are steroid-dependent

**Fig. 7:**
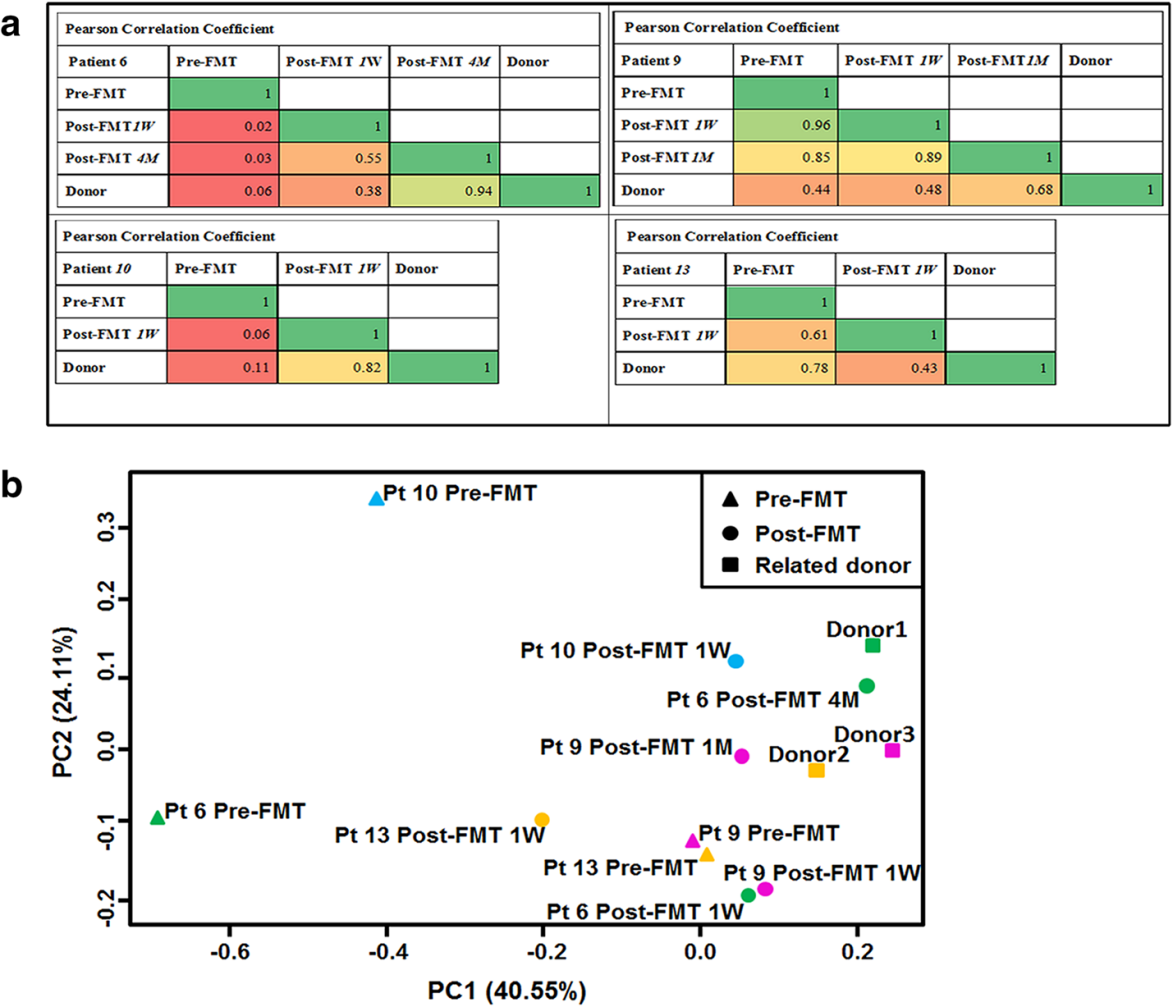
Similarity of fecal microbiota composition at the genus level. **a** Changes in Pearson correlation coefficient at the genus level. Pearson correlation coefficient ranged from 0 (*red*) to 1 (*green*). When the value between two samples is close to 1, the compositions are more similar. **b** Principal coordinate analysis (PCoA) of (Unifrac distance between) fecal microbiota before and after FMT. The distance between the samples represents the similarity of micobiota composition; a closer distance indicates higher similarity. Patient 6 and patient 10 shared the same donor (donor 1)

Analysis of microbiota composition at the phylum (Fig. 5c) and genus (Fig. 6) level indicated significant reconstruction of the bacterial composition following FMT. Except for patient 13 who had used antibiotics, the composition of microbiota in patient 6, 9 and 10 all showed a trend similar to their related donors. The analysis of Pearson correlation coefficient and PCoA indicated the same trend (Fig. 7), especially in patient 6, the Pearson correlation coefficient of the patient-donor comparison at 4 months after FMT was 0.94 (Fig. 7a), which indicated the high similarity of microbiota composition between them.

Patient 13 suffered from perianal abscess due to long-term steroid use, and did not benefit from the first FMT. A thread-drawing surgery was presented at 7 days after the first FMT, and antibiotics were given before and after surgery. The results of our fecal microbiota analyses indicate that use of antibiotic affected the diversity and composition of fecal microbiota greatly.

#### Safety of FMT

There were no severe adverse events during and after FMT procedure. Two patients were observed with fever (less than 39 °C) within 6 h after FMT, and two had transient increase in diarrhea frequency within 24 h after FMT. All symptoms disappeared within 1 day without any medical intervention. One patient had a mild testicular pain after each of two FMTs, which resolved within 12 h.

## Discussion

Our previous study [13] reported the role of intestinal bacterial reconstruction in the treatment of selected refractory CD. This study further explored the role of step-up FMT in steroid-dependent UC. Overall, 57.1 % of recruited patients achieved clinical improvement and sustained steroid independence after step-up FMT and 28.6 % of patients maintained long-term remission (3–18 months). These results indicate that step-up FMT can lead to steroid-free clinical improvement or remission in patients with steroid-dependent UC.

Patients with steroid-dependent UC are considered immunocompromised (IC). Therefore, the use of FMT for steroid dependent patients had been limited due to concerns about safety in this population. Published guidelines recommend avoidance of FMT in solid organ transplant patients [20] and specified that considerations for increased risk in patients on major immunosuppressive agents or patients with severe immunodeficiency [21]. A retrospective study [22] assessed the safety of FMT for immunocompromised patients with CDI which involved HIV/AIDS, solid organ transplant, oncologic condition, immunosuppressive therapy for IBD and other medical conditions or medications. It was reported that while 15 % patients had serious adverse events, none suffered infections which were related to FMT. Brandt et al. [23] published similar results in immunocompromised patients with IBD being treated with FMT. In this study, no severe or obvious adverse events were reported, further strengthening the case that FMT can be performed safely in IC patients. It is noted, however, that in this study steroids were stopped in all the patients at least 1 week before FMT.

A meta-analysis [24] on the FMT in IBD had demonstrated 22 % of patients achieved clinical remission after FMT, while a systemic review [17] concluded that FMT had a success rate close to 90 % in patients with UC. In the present study, 57.1 % of patients achieved clinical improvement and 28.6 % had clinical remission. However, Kump et al. [25] reported that FMT did not induce remission in the patients with chronic active UC, and De Leon et al. [26] reported a patient with UC that had been quiescent for long time who developed a transient flare after treatment of CDI by FMT. The main reason of variable results is unknown, though a recent study by Moayyedi et al. suggests that donor stool composition may play an important role.

We believe that FMT via colonoscopy or enema is suboptimal for patients with extensive UC as they frequently have a difficult time retaining the infused microbiota suspension. In this study, FMT was performed based on a defined volume of purified fecal microbiota according to the established protocol of a standardized lab [13], and the fecal microbiota was delivered into patient’s mid-gut through an esophagogastroduodenoscope.

Three patients who had failed to benefit from the first FMT, improved after the second FMT or after the second FMT plus a short course of steroid treatment. This further suggests that sequential therapy might be an effective strategy for the steroid-dependent UC patients. Weingarden’s study [27] for FMT in the treatment of CDI also showed limited efficacy of single FMT performing for acute CDI, and delaying FMT or performing a second FMT following a course of anti-CDI therapy resulted in better clinical outcomes.

Dysbiosis in patients with IBD reflect a dominance of potentially pro-inflammatory pathogens over anti-inflammatory commensals, which may induce a shift in the immunological balance of the intestinal mucosa toward inflammation [28–30]. It remains unclear whether the dysbiosis of IBD is primarily responsible for the activation of immune system and subsequent inflammation or is a consequence of colitis caused by altered bacterial growth conditions [28, 31]. In this study, a reversal of some of the reported dysbiotic changes in the fecal microbiota was observed in patients after FMT, including marked increase in compositional diversity, decrease in *Proteobacteria*, and the normalization of the *Firmicutes* to *Bacteroidetes* ratio. The four patients whose fecal microbiota was analyzed by deep sequencing achieved clinical improvement or remission after FMT. Two (patient 9 and 13) of them underwent two FMTs due to limited efficacy of the first FMT, and the change of microbiota composition was also limited after the first treatment, while the other two patients (patient 6 and 10) showed obvious response to the first FMT. In all four cases there was obvious restructuring of the intestinal microbiota following FMT. The phenomenon might indicate the degree of microbiota reconstruction was related to the clinical response in the patients with steroid-dependent UC. The major limitation of our study was the missing analysis of microbiota composition from patients who did not respond to FMT and their corresponding donors.

Numerous strategies such as immunomodulators, biologic therapy and surgery [4, 7, 32, 33] are available to induce and sustain remission in steroid-dependent UC. This study suggests that step-up FMT might be an added therapy for this challenging patient population despite the study weaknesses, such as the limited cases, absence of control group, and insufficient endoscopic evaluation for each case during follow-up. Multi-center randomized clinical trials with larger sample size and long-term follow up should be performed to provide a clearer conclusion. Further research is required to explore the mechanisms of step-up FMT, especially understanding the role of restoring microbiota and its effects on host immunity.

FMT provided therapeutic options for patients with IBD; however, not each patient can benefit from single FMT. This study indicated the light of step-up FMT strategy using repeat FMT and steroids for steroid-dependent UC. We hypothesize that step-up FMT strategy should be a concept, which means, FMTs can not only combine with steroids but also other potential therapy, such as anti-TNFα antibody. Actually, based on this step-up strategy, an observational study of using FMT in conjunction with biologics therapies for IBD is ongoing in our center. We will report the results in the future.

## Conclusion

This pilot study reports a novel concept of using the step-up FMT strategy for the patients with steroid-dependent UC. The efficient treatment might be due to the successful reconstruction and maintenance of intestinal microbiota. The present prospective study provides preliminary evidence for supporting our hypothesis that step-up FMT might be a key therapy in the treatment of IBD.

## Additional file

Additional file 1:
**Table S1.** Analysis of laboratory parameters after FMT therapy.
