# Supplementary material for: Step-up fecal microbiota transplantation strategy: a pilot study for steroid-dependent ulcerative colitis
Source: J Transl Med. 2015 Sep 12;13:298. doi: 10.1186/s12967-015-0646-2 (PMC4567790; doi:10.1186/s12967-015-0646-2)
Supplement: Additional file 1: — Table S1. Analysis of laboratory parameters after FMT therapy. [file 12967_2015_646_MOESM1_ESM.docx]

| Table S1. Analysis of laboratory parameters after FMT therapy | | | |
| --- | --- | --- | --- |
| Parameters | **Clinical response to FMT (n)** | **Pre- FMT** | **Post-FMT** |
| CRP (mg/L, mean ±s.d.) |  |  |  |
|  | NR (6) | 35±22.3 | 29.3±25.1 |
|  | CI/ CR (8) | 14.8±16.4 | 5.5±2.3 |
| ESR (mm/h, mean ±s.d.) |  |  |  |
|  | NR (6) | 41.5±17.0 | 45.0±8.2 |
|  | CI/ CR (8) | 25.0±20.4 | 14.2±6.0* |
| WBC (x10^9^/mm^3^, mean ±s.d.) |  |  |  |
|  | NR (6) | 7.2±1.8 | 7.5±1.4 |
|  | CI/ CR (8) | 6.6±2.1 | 5.7±1.4 |
| Lymphocytes subgroup  (%, mean ±s.d.) |  |  |  |
| *T lymphocytes* |  |  |  |
|  | NR (6) | 78.5±8.7 | 75.8±8.9 |
|  | CI/ CR (8) | 72.8±12.8 | 74.4±10.7 |
| *B lymphocytes* |  |  |  |
|  | NR (6) | 13.3±6.8 | 9.7±2.1 |
|  | CI/ CR (8) | 9.9±3.6 | 9.8±3.6 |
| *CD3+ CD4+* |  |  |  |
|  | NR (6) | 40.3±4.5 | 35.9±6.1 |
|  | CI/ CR (8) | 36.7±8.8 | 36.5±8.3 |
| *CD3+ CD8+* |  |  |  |
|  | NR (6) | 36.5±12.6 | 34.1±11.9 |
|  | CI/ CR (8) | 34.0±13.4 | 35.4±13.0 |
| *NK cells* |  |  |  |
|  | NR (6) | 6.4±4.4 | 15.6±16.4 |
|  | CI/ CR (8) | 15.1±11.1 | 14.2±9.1 |
| *CD4+/ CD8+* |  |  |  |
|  | NR (6) | 1.2±0.5 | 1.1±0.2 |
|  | CI/ CR (8) | 1.1±0.2 | 1.2±0.7 |

CRP: C-reactive protein; ESR: erythrocyte sedimentation rate; WBC: white blood cells; NR: non response; CI/CR: clinical improvement or clinical remission.

Parameters between the patients before and post FMT, *Paired t test*, there were no significant difference of all groups.

Parameters of patients with NR to FMT compared with patients achieved CI /CR after FMT, *t test*,* *P*<0.05.
